# Mechanisms and Regulation of the Mitotic Inheritance of the Golgi Complex

**DOI:** 10.3389/fcell.2015.00079

**Published:** 2015-12-16

**Authors:** Carmen Valente, Antonino Colanzi

**Affiliations:** Institute of Protein Biochemistry, National Research CouncilNaples, Italy

**Keywords:** cell cycle, mitosis, golgi complex, mitotic spindle, Arf, Rab

## Abstract

In mammalian cells, the Golgi complex is structured in the form of a continuous membranous system composed of stacks connected by tubular bridges: the “Golgi ribbon.” At the onset of mitosis, the Golgi complex undergoes a multi-step fragmentation process that is required for its correct partition into the dividing cells. Importantly, inhibition of Golgi disassembly results in cell-cycle arrest at the G2 stage, which indicates that accurate inheritance of the Golgi complex is monitored by a “Golgi mitotic checkpoint.” Moreover, mitotic Golgi disassembly correlates with the release of a set of Golgi-localized proteins that acquire specific functions during mitosis, such as mitotic spindle formation and regulation of the spindle checkpoint. Most of these events are regulated by small GTPases of the Arf and Rab families. Here, we review recent studies that are revealing the fundamental mechanisms, the molecular players, and the biological significance of mitotic inheritance of the Golgi complex in mammalian cells. We also briefly comment on how Golgi partitioning is coordinated with mitotic progression.

## Introduction

The Golgi complex is the central organelle in the secretory pathway, and it mediates the modification, sorting and transport of proteins and lipids (De Matteis and Luini, 2008). The structural organization of the Golgi complex varies across organisms. In mammals, it consists of flat cisternae that are grouped into several stacks (the compact zones) that are themselves laterally interconnected by tubular membrane “bridges” (the non-compact zones) to form the “Golgi ribbon” (Sütterlin and Colanzi, 2010). The complex organization of the Golgi ribbon is highly dynamic, and this allows large rates of membrane flux and rapid changes in shape, combined with disassembly and reassembly of the Golgi ribbon under different physiological conditions (Rothman and Wieland, 1996).

For instance, during cell division, these Golgi membranes must be exactly partitioned into the two daughter cells, as for the other organelles and the DNA. This Golgi segregation occurs through regulated and reversible multi-step ribbon disassembly, to produce small fragments that can then be inherited by the daughter cells. This process initiates in the G2 phase of the cell cycle, when the Golgi ribbon is cut into individual Golgi stacks. Then during prophase/prometaphase, these are unstacked and undergo vesiculation, followed by progressive shortening into vesicular/tubular clusters and small fragments. At metaphase, these fragments appear as the “Golgi haze.” At telophase, this haze is gradually reassembled into stacks, to eventually re-form a Golgi ribbon in each of the daughter cells (Shorter and Warren, 2002; Altan-Bonnet et al., 2004; Colanzi and Corda, 2007).

Inhibition of Golgi ribbon unlinking induces a potent and persistent block in the G2 phase of cell-cycle progression (Sütterlin et al., 2002; Hidalgo Carcedo et al., 2004), which is known as the “Golgi checkpoint.” This novel cell-cycle checkpoint is not mediated by activation of the DNA-damage checkpoint; instead, it has been postulated to sense the integrity of the Golgi complex (Sütterlin et al., 2002; Hidalgo Carcedo et al., 2004). Thus, these studies have revealed that Golgi partitioning is an essential and required event for cell entry into mitosis. This highlights the regulatory interplay between organelle inheritance and the signaling pathways that regulate cell division.

Here, we review the current understanding of the mechanisms of Golgi ribbon breakdown in mammalian cells in G2, and we discuss the signaling events that coordinate this Golgi checkpoint.

## Organization of mammalian golgi membranes in interphase

The number of cisternae within a Golgi stack and the number of Golgi stacks in a cell are extremely variable across different species and tissues. In the yeast *Saccharomyces cerevisiae* there is no stacked Golgi structure, and instead the Golgi exists as individual cisternae that are dispersed throughout the cytoplasm (Rossanese et al., 1999). In contrast, in the yeast *Pichia pastoris*, each Golgi stack contains three or four cisternae (Mogelsvang et al., 2003). Up to 30 flattened cisternae per stack have been observed in plants and lower organisms, while mammalian cells can have up to 100 Golgi stacks, each composed of 5–8 cisternae (Beams and Kessel, 1968).

The characteristic structure of the Golgi stack and its ribbon organization in mammals rely on numerous molecular machineries. Among these, the peripheral membrane golgins and the Golgi matrix proteins (i.e., GRASP65, GRASP55) mediate the functional three-dimensional arrangement of the Golgi membranes (Puthenveedu et al., 2006; Feinstein and Linstedt, 2008; Munro, 2011). The Golgi ribbon organization is also under the control of an intact actin and microtubule cytoskeleton, as well as specialized cytoskeleton-based motors and membrane input from the endoplasmic reticulum (ER; Thyberg and Moskalewski, 1999; Rios and Bornens, 2003; Marra et al., 2007). The Golgi complex is also positioned in a perinuclear region near to the centrosome, where it remains due to a microtubule-dependent mechanism (Rambourg and Clermont, 1990).

However, why the Golgi ribbon is organized into stacks of cisternae in interphase remains an important unresolved issue. It is indeed questionable whether cisterna stacking is required for protein transport, particularly considering the efficient secretion in *S. cerevisiae* where the Golgi cisternae are unstacked and dispersed throughout the cytoplasm (Papanikou and Glick, 2009).

Although, the functional significance of the pericentriolar position of Golgi stacks is not completely understood, it has been recently proposed to contribute to: (i) the interactions between the Golgi complex and the centrosome, to facilitate Golgi-mediated centrosome organization and function (Kodani and Sütterlin, 2009); (ii) cell entry into mitosis after Golgi ribbon fragmentation and temporary loss of its pericentriolar position (Sütterlin et al., 2002); and (iii) cell polarization and migration. For example, disruption of the pericentriolar position after Golgin-160 or GMAP210 depletion affects directional protein secretion, which is required for cell migration (Yadav et al., 2009). In addition, increased protein transport rate and glycosylation defects have been shown recently as a result of loss of Golgi stacking (Xiang et al., 2013). This thus, indicates a role for cisterna stacking in the regulation of the accuracy of protein glycosylation.

In conclusion, the ribbon organization, cisterna stacking, and pericentriolar position of the Golgi complex appear to add another level of regulation for these mammalian-cell-specific processes.

## Mechanism of mitotic golgi disassembly

**Figure 1:**
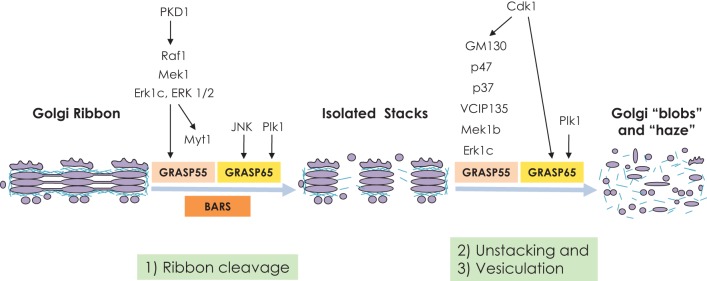
**Schematic representation of mitotic partitioning of the Golgi ribbon**. This first step of mitotic Golgi fragmentation (i.e., ribbon unlinking) controls G2/M transition and requires the activities of BARS, GRASP55, and GRASP65. These processes also require the activities of several MAP kinase components, as indicated. At the onset of mitosis, these isolated stacks undergo further disassembly in the sequential steps of unstacking and vesiculation. These lead to the formation of the so-called “Golgi haze” during metaphase, where the Golgi membranes are completely fragmented. These processes require the activities of the kinases Plk1 and Cdc2, and their targets GRASP55 and GRASP65. Subsequent events result in dephosphorylation of the kinases that took part in the initial fragmentation, which leads to reassembly of the Golgi ribbon in the daughter cells.

Mitotic segregation of the Golgi complex involves progressive and reversible disassembly of the Golgi ribbon into dispersed fragments (Figure 1), to allow correct partitioning of the Golgi membranes between daughter cells (Shorter and Warren, 2002; Colanzi et al., 2003; Altan-Bonnet et al., 2004). A number of the molecular players here have been identified and characterized through two biochemical approaches: semi-intact cell assays, and Golgi mitotic disassembly/reassembly assays (Tang et al., 2010; Colanzi and Sütterlin, 2013). These two assays have provided a highly manipulable approach, within which the sequence of morphological events can be precisely followed by electron microscopy or biochemical analysis.

### A. severing the golgi ribbon into stacks

The first step of mitotic Golgi disassembly occurs in the G2 phase and consists of the fragmentation of the non-compact zones of the Golgi ribbon. Then, at the onset of mitosis, these isolated Golgi stacks are converted into scattered tubule-reticular elements that are further fragmented and dispersed throughout the cytoplasm (Figure 1). The cleavage of these membrane tubules is now known to be necessary and sufficient for cell entry into mitosis (Colanzi et al., 2007; Feinstein and Linstedt, 2007). Several crucial factors involved in this mitotic Golgi disassembly have been identified. Among these, there are the fission-inducing protein CtBP1-S/BARS (referred to here as BARS; Hidalgo Carcedo et al., 2004) and the peripheral Golgi proteins GRASP65 and GRASP55 (Sütterlin et al., 2002; Xiang and Wang, 2010). Each of these proteins has a specialized role in Golgi-ribbon unlinking, and their combined activities drive the cleavage of the Golgi interstack connections in late G2 phase of the cell cycle. Roles for these proteins in the further fragmentation of the isolated Golgi stacks into the “Golgi haze” that is detected in metaphase have also been shown (Figure 1).

#### BARS

BARS is a protein with a dual role, as it can act as a transcriptional co-repressor in the nucleus, and as a regulator of Golgi tubule fission in membrane trafficking (Hidalgo Carcedo et al., 2004; Colanzi et al., 2007). BARS controls mitotic disassembly of the Golgi stacks by severing the tubular network of the non-compact zones (Colanzi et al., 2007). The functional role of BARS in mitosis has been studied both *in vitro* and *in vivo*. *In vitro*, both the depletion and inhibition of BARS strongly inhibit mitotic Golgi fragmentation. *In-vivo* studies using fluorescence recovery after photobleaching have defined the role of BARS in the fission of the interstack connecting tubules, which is a crucial step for G2/M cell-cycle transition (Colanzi et al., 2007). However, interference with BARS activity in interphase does not lead to Golgi fragmentation (Hidalgo Carcedo et al., 2004), which indicates that BARS is specifically activated in G2 phase to promote the severing of the Golgi ribbon. It is reasonable that this activation is mediated by a specific BARS phosphorylation event that occurs in the G2/M phases, as has been shown for BARS activation in membrane traffic (Liberali et al., 2008; Valente et al., 2012, 2013). BARS phosphorylation might induce its binding to specific partners that then promote Golgi ribbon fission. Moreover, BARS-knockout fibroblasts have their Golgi ribbon divided into isolated stacks at all cell-cycle stages, which bypasses the requirement for BARS for G2/M Golgi ribbon unlinking (Colanzi et al., 2007).

#### Golgi reassembly and stacking proteins (GRASPs)

The mitotic functions of the Golgi reassembly stacking proteins (hence GRASPs) GRASP65 and the structurally related GRASP55 are better defined. Both GRASP65 and GRASP55 are peripheral membrane proteins that have two N-terminal PDZ-like domains and a C-terminal serine/proline-rich regulatory domain (Vinke et al., 2011). The localization of GRASP65 to the *cis*-Golgi and of GRASP55 to the *medial/trans* Golgi is mediated by their N-terminal myristic acid (the GRASP domain) and their binding to specific golgins (Vinke et al., 2011). Both GRASP65 and GRASP55 are essential factors for formation and maintenance of the tubules that connect the Golgi stacks (Sütterlin et al., 2005; Puthenveedu et al., 2006; Feinstein and Linstedt, 2008). Depletion of GRASP65 and GRASP55 induces Golgi ribbon unlinking and a reduction in the number of cisternae per stack (Sütterlin et al., 2005; Puthenveedu et al., 2006; Feinstein and Linstedt, 2008; Jarvela and Linstedt, 2014). Moreover, inhibition of the activities of GRASP65 and GRASP55 alters mitotic Golgi fragmentation and mitotic entry (Sütterlin et al., 2002; Preisinger et al., 2005; Duran et al., 2008).

#### GRASP65

GRASP65 is phosphorylated at multiple sites in its C-terminal serine/proline-rich domain during mitosis, by Cdk1 and Plk1 kinases (Lin et al., 2000; Wang et al., 2003; Preisinger et al., 2005; Sütterlin et al., 2005; Yoshimura et al., 2005). *In-vitro* studies have shown that the GRASP proteins form homo-oligomers in *trans*, and these interactions are prevented by phosphorylation of specific residues within the C-terminal GRASP65 serine/proline-rich domain (Tang et al., 2010). Cdk1 targets GRASP65 at four serine/threonine residues (i.e., Ser216/Ser217, Thr220, Ser277, Ser376). Accordingly, mutation of these sites into alanines prevents mitotic Golgi unlinking. Plk1 has been shown to dock onto Cdk1-phosphorylated GRASP65 and then to phosphorylate GRASP65 at Ser189. This Cdk1-mediated and Plk1-mediated phosphorylation abrogates the *trans*-oligomerization properties of GRASP65. The consequence of this is prevention of its tethering function for the connection of the cisternae in the formation of the stacked Golgi, and its linking of adjacent stacks to form the Golgi ribbon (Wang et al., 2003, 2005; Sengupta and Linstedt, 2010). More recently, it has been shown that the kinase JNK2 is a potent and crucial regulator of Golgi unlinking during G2. This action of JNK2 is mediated by phosphorylation of Ser277 of GRASP65, which reveals a JNK2-GRASP65 signaling axis that couples Golgi inheritance and G2/M transition (Cervigni et al., 2015). Altogether, these data suggest a link between the C-terminus phosphorylation events on GRASP65 and mitotic Golgi fragmentation. This hypothesis has been recently validated by studies of the conformational changes in the crystal structure of the GRASP65-PDZ domain under Plk1-mediated phosphorylation, which alters the membrane tethering function of GRASP65 (Truschel et al., 2012). Moreover, injection of an affinity-purified blocking anti-GRASP65 antibody (which recognizes its C-terminal region) into mitotic cells inhibits the reforming of the stacks and of the Golgi ribbon after cell division (Wang et al., 2003).

#### GRASP55

Similar to GRASP65, GRASP55 is phosphorylated during mitosis. ERK2 is a downstream target of mitogen-activated protein (MAP) kinase kinase 1 (MEK1) and it phosphorylates GRASP55, which has been shown to be required for Golgi ribbon unlinking and mitotic progression (Duran et al., 2008; Feinstein and Linstedt, 2008; Xiang and Wang, 2010). Of note, expression of a MEK1/ERK2-mediated phospho-defective mutant of GRASP55 (T222, 225A) prevents Golgi fragmentation and the consequent G2/M cell-cycle transition (Feinstein and Linstedt, 2008). This MEK1/ERK2/GRASP55-mediated G2/M block/delay is rescued in GRASP65-depleted cells where the Golgi ribbon is already fragmented (Feinstein and Linstedt, 2008). These data indicate that GRASP65 and GRASP55 cooperate in Golgi fragmentation and mitotic progression.

### Key regulatory pathways

#### PKD/RAF/MEK/ERK/GRASP55

The RAF1/MEK/ERK signaling pathway is the best characterized kinase cascade in G2/M unlinking of the Golgi ribbon (Acharya et al., 1998; Feinstein and Linstedt, 2007). MEK1 is first mitotically activated by RAF1, and then recruited to the Golgi ribbon in late prophase, where it mediates Golgi complex fragmentation (Colanzi et al., 2003). Accordingly, blocking MEK1 function by RNA interference (RNAi) or using the MEK1/2-specific inhibitor U0126 prevents ribbon unlinking, which inhibits cells entry into mitosis (Feinstein and Linstedt, 2007). The activation of RAF1/MEK1 in mitosis is also under the control of PKD1 and PKD2 (Kienzle et al., 2013), which are key factors in the regulation of post-Golgi carrier formation (Yeaman et al., 2004; Valente et al., 2012). Interfering with PKD activity blocks mitotic activation of RAF1 and MEK1, and, as a consequence, ribbon unlinking is blocked, and the cells accumulate in the G2 phase (Kienzle et al., 2013). Of note, this phenotype is recovered by expression of active MEK1, which indicates that PKD is involved in the same RAF1/MEK1 regulatory Golgi partitioning pathway (Kienzle et al., 2013). Among the downstream MEK1 substrates, the best characterized is ERK1/2, which phosphorylates GRASP55 to induce mitotic Golgi breakdown (Feinstein and Linstedt, 2008). ERK1c is the second MEK1 effector, which is a splice variant of ERK1 that is highly expressed, phosphorylated and activated in mitosis. ERK1c is activated by MEK1b-mediated phosphorylation. Modulation of ERK1c endogenous levels strongly affects mitotic Golgi fragmentation (i.e., overexpression, increased fragmentation; depletion, impaired fragmentation; Shaul and Seger, 2006). In addition, Plk3 (Polo like kinase 3) has been identified as another Golgi-localized downstream target of MEK1/ERK kinases (Ruan et al., 2004). Plk3 activation induces Golgi ribbon fragmentation (Ruan et al., 2004), and in embryonic fibroblasts from Plk3 knock-out mice, this fragmentation is partially inhibited (Xie et al., 2004). Furthermore, Plk3 binds, phosphorylates and activates VRK1. Inhibition of VRK1 function hampers MEK1-induced Golgi fragmentation (López-Sánchez et al., 2009). However, for these Plk3, VRK1, and ERK1c actions in the severing of the Golgi ribbon and in the conversion of mini-stacks into “Golgi blobs,” their specific contributions and Golgi targets remain unknown. Finally, the protein kinase Myt1 is an important MEK effector, as it negatively regulates Cdk1 activity and is associated with the Golgi complex and the ER (Liu et al., 1997). RNAi studies in *Drosophila* and HeLa cells have revealed a requirement for Myt1 in mitotic Golgi dynamics (Cornwell et al., 2002; Nakajima et al., 2008). In addition, a recent study reported that Myt1 depletion causes accelerated entry into mitosis and increased fragmentation of the Golgi complex during G2 (Villeneuve et al., 2013). The hypothesis that Myt1 functions downstream of MEK1 was suggested by the finding that Myt1 is inactivated by MEK1-mediated phosphorylation, and that Myt1 depletion bypasses the requirement for MEK1.

#### Plk1/GRASP65 and JNK2/GRASP65 pathways

Plk1 is one of the key actors in mitotic Golgi fragmentation (Sütterlin et al., 2001; Sengupta and Linstedt, 2010). Plk1 phosphorylates GRASP65 on Ser189, which is part of the N-terminal domain that mediates *trans*-oligomerization of GRASP65 (Truschel et al., 2012). Mutation of this residue to aspartic acid inhibits the GRASP65-mediated tethering function, while alanine substitution hampers mitotic Golgi unlinking (Sengupta and Linstedt, 2010). A model can be hypothesized in which the C-terminal domain of GRASP65 is first phosphorylated by Cdk1, to create a Plk1 binding site. Then GRASP65 is phosphorylated by Plk1 on Ser189, which inhibits the *trans*-oligomerization of GRASP65. However, as the activity of Cdk1 in G2 is low, it is possible to speculate that in addition to Cdk1, other kinases might phosphorylate GRASP65 to generate a Plk1 binding side. Moreover, it has been recently shown that during the G2 phase, GRASP65 is specifically phosphorylated in Ser227 by JNK2 kinase (Cervigni et al., 2015). The blocking of JNK2 kinase function by RNAi or using three unrelated JNK inhibitors persistently blocks the cell cycle in G2 phase. This study led to a model in which the JNK2/GRASP65 pathway is a crucial linker of mitotic Golgi ribbon partitioning with G2/M phase transition (Cervigni et al., 2015).

### B. disassembly of golgi stacks into “Blobs” and “Haze”

The further fragmentation of the isolated Golgi stacks starts in prophase and results in dispersed “Golgi blobs” and “Golgi haze” throughout the cytoplasm (Misteli and Warren, 1995). This additional fragmentation step depends on the unstacking of the Golgi membranes, followed by an additional vesiculation step. Golgi cisterna unstacking requires Cdk1-mediated phosphorylation of GRASP65 and GRASP55, and thereby inhibition of their formation of *trans*-oligomers. In cell-free systems, mitotic disassembly of Golgi cisternae occurs concomitant to unstacking, and can follow two distinct pathways. One pathway proceeds via COPI vesicles, which continue to bud from the Golgi stack (Sönnichsen et al., 1996) without tethering to their target membrane (Shorter and Warren, 2002). Concomitantly, a COPI-independent pathway breaks up the flattened cisternae into tubular-vesicular fragments (Shorter and Warren, 2002). The nature of these fragments and the mechanisms of their inheritance in the two daughter cells have been described according to two opposing models.

The first model suggests that the Golgi complex is in dynamic equilibrium with the ER, and thus a block of membrane transport in mitosis would induce Golgi membrane disassembly (Zaal et al., 1999; Altan-Bonnet et al., 2004; Rhee et al., 2005). The inactivation of two small GTPases that operate at the ER/Golgi interface, Arf1 and Sar1, would mediate this effect (Altan-Bonnet et al., 2004). In prophase, Cdc2-mediated inactivation of Sar1 would reduce the export of membranes and proteins from the ER (Prescott et al., 2001; Kano et al., 2004). As a consequence, the membrane transport cycle between the Golgi complex and the ER would be impaired, which would induce progressive redistribution of Golgi membranes into the ER. The rate of this accumulation would be further enhanced by increased protein transport from the Golgi to the ER in the initial phases of mitosis, after dissociation of Arf1 from the Golgi membranes (Altan-Bonnet et al., 2004). This model finally suggests that the Golgi is inherited together with the ER (Zaal et al., 1999), and at mitotic exit, the restarting of membrane transport would re-establish normal interphase Golgi structure and function (Altan-Bonnet et al., 2004).

The second model suggests that the mechanism of Golgi inheritance is achieved in an ER-independent manner. Thus, it indicates that the Golgi complex is an autonomous organelle that forms the mitotic Golgi remnants required for post-mitotic reassembly (Barr, 2004). In this case, the key event of Golgi inheritance is the disruption of the membrane tethering complexes by mitotic-activated kinases (Shorter and Warren, 2002). As a result, the Golgi stacks are fragmented into tubular-vesicular clusters and the small vesicles of the Golgi haze, which are then actively partitioned between the two daughter cells in a mitotic-spindle-mediated process (Shima et al., 1998; Seemann et al., 2002; Axelsson and Warren, 2004; Barr, 2004; Puri et al., 2004). According to this model, at the end of mitosis, inhibition of mitotic kinases would reverse this series of events to allow the Golgi to be reassembled.

Many pieces of evidence support the second ER-independent Golgi partitioning model. Indeed, it has been shown that mitotic Golgi fragments accumulate near the spindle poles, while the ER is not present in the spindle area (Puri et al., 2004). Additionally, Seemann and co-workers provided evidence in favor of spindle-dependent Golgi ribbon inheritance (Wei and Seemann, 2009). Indeed, through induction of asymmetric cell division where the spindle was inherited by only one of the daughter cells, they demonstrated that the Golgi reassembled into a ribbon only in the cell with the inherited spindle. Conversely, in the spindle-deprived cell, the Golgi stacks remained dispersed throughout the cytoplasm (Wei and Seemann, 2009). Interestingly, the injection of Golgi protein extracts into these spindle-deprived cells with the addition of purified spindle proteins promoted the reforming of an intact Golgi ribbon (Wei and Seemann, 2009). Thus, the accuracy of Golgi inheritance suggests the involvement of an active and regulated partitioning mechanism, instead of a stochastic process (Shima et al., 1998).

### Molecular mechanism of post-mitotic golgi reassembly

*In-vitro* cisternal regrowth assays from mitotic Golgi fragments have identified NSF (N-ethylmaleimide-sensitive factor) and p97 as two key factors in post-mitotic Golgi reassembly (Acharya et al., 1995; Rabouille et al., 1995). NSF and p97, which is an NEM-sensitive protein homologous to NSF (and is also known as VCP; valosin-containing protein), are ATPases that are associated with a variety of cellular activities of the AAA family of proteins (Brunger and DeLaBarre, 2003). As ATPases, NSF and p97 have specific co-factors: NSF-mediated fusion requires the proteins SNAPs and p115, while the p97-mediated pathway requires formation of a complex with p47 and VCIP135, or with p37 (Kondo et al., 1997; Uchiyama et al., 2006). In the post-mitotic Golgi-reassembly process, two sequential events have been suggested: first, membrane fusion induced by NSF produces large vesicles and tubular-reticular elements, which then fuse to generate cisternae through a p97-mediated process. During mitosis, Cdk1 phosphorylates p47, p37 and VCIP135, and blocks p97-controlled membrane-fusion processes so that the Golgi membranes remain disassembled (Uchiyama et al., 2003; Kaneko et al., 2010; Totsukawa et al., 2013). Thus, mitotic phosphorylation of the membrane fusion machinery can explain the mitotic Golgi phenotype of dispersed tubular-reticular membranes and vesicles in the cytosol.

Additionally, ubiquitination has been shown to have an important role in post-mitotic Golgi reassembly. P47, the adaptor protein of p97, contains a UBA ubiquitin binding domain. P97 binding promotes p47 binding to ubiquitinated proteins via its UBA domain, and this domain is necessary for Golgi reassembly (Meyer et al., 2000). VCIP135 (valosin-containing protein p97/p47 complex–interacting protein; p135) is a positive factor for p97/p47-mediated membrane fusion (Uchiyama et al., 2002). Indeed, it has been shown to have a deubiquitinase activity, which was necessary for Golgi reassembly (Zhang and Wang, 2015). In early mitosis, Cdk1-mediated phosphorylation on Ser130 inhibits the deubiquitinase activity of VCIP135, and this inactivates p97/p47-mediated Golgi membrane fusion. Conversely, at the end of mitosis, VCIP135 is dephosphorylated (on Ser130), and this is associated with recovery of its deubiquitinase activity and with post-mitotic Golgi reassembly (Zhang and Wang, 2015). In addition to the stimulation of the fusion processes, dephosphorylation of the GRASP proteins favors the stacking and linking of the reformed Golgi cisternae, thus leading to the reassembly of the Golgi complex after mitosis.

In conclusion, the Golgi ribbon can be viewed as a metastable structure that is maintained by several core and accessory proteins, and that can rapidly adapt to stressful or physiological conditions.

## Coordination of golgi fragmentation with the cell cycle

### A. why is the severing of the golgi ribbon necessary for G2/M transition?

It is now well-accepted that entry of cells into mitosis requires Golgi fragmentation (Sütterlin et al., 2002; Hidalgo Carcedo et al., 2004). Part of the signaling through which a block in Golgi fragmentation prevents cell-cycle progression has been identified. As the block of Golgi fragmentation induces G2 arrest, it is likely to involve cyclin-B-dependent kinase 1 (CycB-Cdk1), because CycB-Cdk1 is the major regulator of G2/M transition (Nigg, 2001). The activity of CycB1-Cdk1 activity is known to be controlled by recruitment and activation of the Ser/Thr kinase Aurora-A at the centrosome (Marumoto et al., 2002). Interestingly, a block in Golgi fragmentation interferes with Aurora-A recruitment to the centrosome in G2, and prevents its activation (Persico et al., 2010). This link between Golgi organization in G2 and Aurora-A recruitment and activation provides the first mechanistic insight into how Golgi dynamics can be coordinated with cell-cycle progression.

However, a second cyclinB-dependent, but Aurora-independent, mechanism might also contribute to Golgi-mediated control of mitotic entry. Mammalian cells express two B-type cyclins, CycB1 and CycB2, and CycB2 associates with the Golgi complex and the ER (Jackman et al., 1995). Both CycB1 and CycB2 cooperate to promote mitotic entry. It is possible that activation of the Golgi-associated CycB2-Cdk1 complex is regulated by inhibition of Golgi-localized Myt1 (Villeneuve et al., 2013). More investigations are required to address this issue.

### B. GTPases control the release of golgi-associated proteins during mitosis

There is evidence that complete disassembly of the stacks is not required for mitotic progression (Uchiyama et al., 2003). Also, the second step of Golgi fragmentation in mammals (i.e., disassembly of isolated stacks) is involved in events that are necessary for cell-cycle progression. Indeed, during prophase, disassembly of the Golgi stacks correlates with release of Arf1 and a set of peripheral proteins from the Golgi membranes (Altan-Bonnet et al., 2003). Arf GTPases are key regulators of membrane traffic and organelle structure, and they act through regulation of the recruitment of a large number of effectors. These include components of vesicular coats, membrane tethers, and lipid-binding or lipid-modifying enzymes (Cherfils, 2014). Spatial and temporal regulation of the Arf proteins is through their guanine-nucleotide-exchange factors (GEFs) and GTPase-activating proteins (GAPs), which catalyze GTP binding and hydrolysis, respectively (Cherfils, 2014).

The mechanism through which Arf1 is released from Golgi membranes has started to emerge more recently. Indeed, a guanine nucleotide exchange factor for Arf that resides at the *cis*-Golgi, GBF1, is a key Arf regulator. During G2/M transition, GBF1 is phosphorylated by AMPK, which results in disassociation of GBF1 from Golgi membrane. Then during mitosis, GBF1 is also phosphorylated by CDK1. This results in the release of Arf from the Golgi membranes, but surprisingly, this does not cause the release of COPI, one of the main Arf effectors for membrane traffic (Morohashi et al., 2010). Phosphorylation of AMPK and GBF1 is essential for Golgi disassembly and subsequent mitosis entry. Active AMPKα (which is phosphorylated on Thr172) transiently localizes to the Golgi complex during late G2/early prophase. Functional inhibition of AMPKα delays G2/M transition in HeLa cells. Furthermore, it has been shown recently that AMPKα2 is activated by CaMKKβ-mediated phosphorylation during late G2 (Lee et al., 2015).

Golgin160 is among the key Golgi-located Arf effectors, and it recruits the dynein motor to regulate the position of the Golgi complex (Yadav et al., 2012). The minus-end-directed motor is required to confer centripetal motility to membranes. During cell division, the association of the dynein motor with membranes is regulated by dissociation of the receptor–motor complex from the membranes, which can explain the dramatic changes in organelle positioning observed during mitosis (Yadav et al., 2012).

An additional Golgi-located regulator of GTP-binding proteins is the non-catalytic β subunit of RalGAPα1/2 β (GTPase activator for RALA/B). RalGAPβ localizes to the Golgi complex and nucleus during interphase, and relocalizes to the mitotic spindle during mitosis. Depletion of RalGAPβ causes chromosome misalignment and decreases the levels of cyclin B1 (Personnic et al., 2014).

Proteins of the Rab family have also shown to have links to Golgi-based processes and mitotic progression. Rab6A' regulates retrograde transport from late endosomes via the Golgi to the ER, and it is involved in the transition from metaphase to anaphase during mitosis. Bicaudal-D1 is an important effector of Rab6B (Wanschers et al., 2007). Rab6B and Bicaudal-D1 co-localize at the Golgi complex and on vesicles, where they regulate the association of these membranes with microtubules. Knocking down Rab6A′ reduces the endocytic intracellular transport of Shiga toxin and causes defects to Golgi-associated protein recycling through the ER. When the function of Rab6A′ is altered, cells are blocked in metaphase with the Mad2-spindle checkpoint activated, which suggests that Rab6A′ participates in a pathway that is involved in metaphase/anaphase transition (Miserey-Lenkei et al., 2006). Rab11 is localized at the *trans*-Golgi network and late endosomes during interphase. During mitosis, Rab11 localizes to the mitotic spindle and regulates dynein-dependent endosome localization at the mitotic spindle poles (Hehnly and Doxsey, 2014). Rab11 depletion prevents localization of recycling endosomes at the spindle poles, delays mitotic progression, disrupts spindle-pole protein recruitment and astral microtubule organization, and alters mitotic spindle orientation. This suggests that Rab11 is a crucial regulator of correct spindle-pole function during mitosis.

Altogether, these findings indicate that during mitosis, and concomitant with the disassembly of Golgi stacks, a series of GTP-binding proteins regulate the changes in the localization and function of Golgi-localized proteins, to regulate the key mitotic events. This suggests that Golgi disassembly is coordinated with chromosome segregation.

Related to this hypothesis, the Golgi-associated protein p115 has been shown to dissociate from the Golgi complex during mitosis. Instead, p115 partitions with the poles of the mitotic spindle, where it controls spindle formation and chromosome segregation and cytokinesis (Radulescu et al., 2011). Furthermore, dissociation of p115 upon mitotic entry favors the interaction of the golgin GM130 with importin α, via a nuclear-localization signal. This interaction leads to sequestration of importin α and liberates the spindle assembly factor TPX2, which can then activate Aurora-A kinase and stimulate local microtubule nucleation. At the spindle, microtubule nucleation is also favored by GM130, thus functionally linking Golgi membranes to spindle formation, which is crucial for faithful chromosome inheritance (Wei et al., 2015).

As a further example of a functional link between Golgi fragmentation and spindle formation, Miki is a Golgi-associated protein that is relocated during mitosis to the centrosome. This occurs through tankyrase-mediated PARsylation and controls the recruitment of CG-NAP, a scaffold protein of the γ-tubulin ring complex (Ozaki et al., 2012). Thus, Golgi vesiculation can be explained by the need to change the subcellular distribution of proteins that acquire specific and novel functions during mitosis.

### C. the role of the golgi reformation during telophase

The Golgi membranes begin to reassemble during telophase, at which time they form two distinct ribbons on the opposite sides of the nucleus in each daughter cell (Altan-Bonnet et al., 2006; Goss and Toomre, 2008). The larger Golgi ribbon is positioned next to the centrosome, while the smaller one is in close proximity to the midbody. During cytokinesis, the smaller Golgi ribbon migrates to the opposite side of the nucleus and fuses with the larger Golgi ribbon. Overall these data suggest that a functional Golgi ribbon might be required to establish asymmetric cell division.

## Conclusions

In conclusion, mitotic partitioning of the Golgi complex is composed of individual and consecutive steps that control different aspects of cell division (Figure 1). G2-specific fragmentation of the Golgi ribbon into isolated stacks is necessary for cell entry into mitosis. Then, during mitosis, selected Arf and Rab GTPases regulate the disassembly of the isolated stacks and the relocation of Golgi-associated proteins. This regulates the events that are necessary for successful cell duplication, such as spindle formation and chromosome segregation.

Further investigations into the Golgi fragmentation process and the molecular players involved in the control of the different stages of mitosis will contribute to better understanding of the mechanisms by which the disassembly of the Golgi complex controls G2/M transition and mitosis. These will have the potential to reveal novel targets and approaches for pharmacological intervention in many diseases, including cancers.

## Author contributions

CV and AC wrote the manuscript.

### Conflict of interest statement

The authors declare that the research was conducted in the absence of any commercial or financial relationships that could be construed as a potential conflict of interest.

## Abbreviations

Arf: ADP-ribosylation factor
Cdk1: Cyclin dependent kinase 1
CtBP1-S/BARS, C-terminal-binding protein 1: short form/brefeldin-A-dependent ADP-ribosylation substrate
ERK1: extracellular-signal-regulated kinase 1
GRASP: Golgi complex reassembly stacking protein
MEK1: mitogen-activated protein kinase kinase 1
Plk: Polo-like kinase
Rab: Ras-related GTP-binding protein
Raf1: recombinase-activating factor 1
VRK1: vaccinia related kinase 1.
